# Green Chemometric—assisted UV spectrophotometric techniques for the analysis of *Helicobacter pylori* triple therapy: amoxicillin, metronidazole and famotidine in bulk, individual and laboratory- prepared combined dosage forms: application to simulated gastric fluid with comprehensive greenness and whiteness appraisals

**DOI:** 10.1186/s13065-025-01387-4

**Published:** 2025-02-19

**Authors:** Hadir M. Maher, Hoda Mahgoub, Marwa A. A. Ragab, Salma Tarek

**Affiliations:** Department of Pharmaceutical Analytical Chemistry, Faculty of Pharmacy, Alexandria University, Elmessalah, Alexandria, 21521 Egypt

**Keywords:** Chemometric-UV spectrophotometric methodologies, First derivative and double divisor ratio spectra, Fourier function based on absorbance data, *Helicobacter pylori* triple therapy

## Abstract

A significant variable in the genesis of peptic ulcers is *Helicobacter pylori*, which could be eliminated through several strategies. Amid them, there is a strategy based on receptor histamine-2 (e.g., famotidine) antagonist in conjunction with metronidazole and amoxicillin as antibiotics. This research used UV spectrophotometry paired with chemometrics to clarify the concurrent analysis of famotidine (FAM), amoxicillin (AMX) and metronidazole (MET) in bulk powder and mixed tablet formulations. The first derivative was calculated for determining FAM and MET. Nonetheless, the methods of double divisor ratio spectra and Fourier functions were incorporated for determining each of the three medications. The methods linearity was fine in the strength ranges of 3–20 µg mL^−1^ (FAM), 12–40 µg mL^−1^ (AMX) and 4 -20 µg mL^−1^ (MET), as shown by the correlation coefficient (0.9999). Analyzing the triple therapy of FAM, AMX and MET helped in the determination of the techniques' accuracy and precision which were done for both their synthetic and combined tablet mixture solutions, as well as in their combined tablet mixture solutions in simulated stomach fluid. Adopting UV spectrophotometry was advantageous over HPLC and HPTLC techniques for FAM, AMX and MET simultaneous determination. This can be attributed to better cost and time efficiency, lack of sophistication, less amounts of reagents and wastes and therefore better greenness when talking about UV spectrophotometry. Upon co-formulation of FAM, AMX and MET triple therapy, their routine analysis could benefit from the presented methodologies in quality control labs. Green Analytical Chemistry (GAC) as well as White Analytical Chemistry (WAC) were both employed for assessing the UV spectrophotometric methods that were described with an emphasis on the cost-effectiveness and environmental friendliness of the suggested techniques. Moreover, the method was practically appraised employing Blue Applicability Grade Index (BAGI).

**Supplementary Information:**

The online version contains supplementary material available at 10.1186/s13065-025-01387-4.

## Introduction

*Helicobacter pylori* (*H.pylori*) which falls within the Gram- negative organisms, colonizes in the stomach. It can use acid resistant mechanisms to get through this extreme environment. Although reports indicate that this virus is more common in underdeveloped nations, the overall incidence of this infection varied among nations. This bacteria is significant not only because it causes digestive health conditions (chronic gastritis, peptic ulcers, and gastric cancer) and affects about 50% of the global population, but additionally because it is linked to extra-gastric disorders which demand more research [1, 2]. As a result, its bacterial eradication treatment is still of interest and different procedures or protocols have been proposed. One of these procedures is the triple therapy consisting of amoxicillin (AMX), metronidazole (MET), and famotidine (FAM).

This triple therapy is the target of the presented work to analyze and determine its components using different spectrophotometric techniques. PPIs, also known as proton pump inhibitors, or histamine-2 receptor antagonists (H2RAs), such as FAM, are often given with varyig doses of AMX and MET in an effort to cure H. pylori. Different protocols are set up: for example,40 mg FAM is taken with AMX (500 mg, 750 mg or 1000 mg, twice (b.i.d), thrice (t.i.d) or four times (q.i.d) a day) and MET (250 mg or 500 mg, b.i.d, t.i.d or q.i.d) for 7 to 14 days as a triple therapy for *H.pylori* in case of Clarithromycin or Levofloxacin resistance [3–6]. Both H2RAs and PPIs are similarly successful at eliminating H. pylori, however the former is less expensive than the latter [4, 7].

AMX is an extended range penicillin and MET is a synthetic nitroimidazole derivative with antiprotozoal and antibacterial activities while FAM is an H2RA [8]. Suppl. Fig. S1 illustrated the chemical structures of the three drugs under investigation [9, 10].

UV spectrophotometry is broadly used for the drugs quantitation either in bulk or in pharmaceutical products. This is attributed to its simplicity, precision, reliability, minimum solvent usage and short analysis time [11]. However, the broad UV spectra hinder the analysis of multi-component mixtures which needs further data handling for resolving mixtures.

Derivative spectrophotometry is a straightforward and uncomplicated analytical technique used to overcome the problem of overlapping spectra upon resolving mixtures without the need for preceding separation steps [12, 13].

The zero – crossing derivative method is fast, simple and precise. Zero-crossing point of the derivative spectrum of one of two components is chosen to carry out measurements which would be a function of concentration of the other component alone [14]. This method permitted the determination of different binary[15] and ternary mixtures[16]. However, if no zero crossing points are found, the derivative could be optimally substituted by the ratio derivative technique.

Ratio derivative spectroscopy is also a useful technique which was introduced by Salinas et al., 1990 [17]. It is carried out by dividing the overall spectrum of a binary mixture by the spectrum of one of the two components then the resultant spectrum is the ratio spectrum of the second component. Once the first derivative of this ratio spectrum is then determined, the amplitude of the signals obtained will be corresponding to the concentration of the second component in the mixture solution. Similarly, using the second component as a divisor is able to offer information about the concentration of the first one [18]. This method can also be adopted for the determination of the ternary mixtures using a double divisor as first illustrated by Dinc et al. [19, 20]. By dividing the ternary mixture's absorption spectra by the total of its two components' absorption spectra, the ratio spectra are found using this method. By choosing the points of coincidence, maximum and/or minimum, between the mixture and the mentioned component—so-called derivative double divisor ratio spectra, the remaining third component could subsequently be found using the first derivative of the ratio spectra [21, 22].

Another promising technique for resolving mixtures is using Fourier transform to process absorbance spectra which aims to eliminate different types of interferences and solve problems such as overlapping spectra or low concentrations of certain analytes in an analytical mixture. Fourier functions have numerous applications in analytical chemistry as they have been applied to diverse techniques including spectrophotometry [23], high performance liquid chromatography [24] and spectrofluorimetry [25].

It is important to understand that literature has discussed the determination of AMX, MET and FAM in their ternary mixture using HPLC [26]. Furthermore, the literature revealed that AMX and MET were simultaneously determined in their mixture with other four antimicrobials and three proton pump inhibitors using a chromatographic method with DAD [27]. The authors have published a technique based on utilizing HPTLC for the simultaneous determination of the three afore-mentioned drugs [28] and another technique utilizing HPLC for the simultaneous determination of vonoprazan (VPZ), AMX and MET [29] in bulk powder and simulated gastric juice not long ago. Moreover, AMX and MET were determined in their binary mixture using hydrophilic interaction chromatography (HILIC) [30], UV spectroscopy [31, 32], and HPLC [33]. However, AMX, MET, and FAM haven´t been simultaneously determined in their ternary mixture using UV- spectrophotometric methods.

The suggested chemometric-UV spectrophotometric technique proved to be advantageous over reported HPLC [26] and HPTLC [28] techniques for FAM, AMX and MET simultaneous determination. This can be attributed to better cost and time efficiency, lack of sophistication, less amounts of reagents and wastes and therefore better greenness when talking about UV spectrophotometry.

This work elucidated three divergent economic, fast, green and simple spectrophotometric methods for simultaneous analysis of AMX, MET and FAM ternary mixture in bulk and laboratory prepared combined tablet mixtures in methanol and in simulated gastric fluid without preliminary separation. The main issue that was faced throughout the analysis of such combination was linked to the spectral overlapping of drugs in mixture with high dose of AMX (500 mg tablet) and MET (250 mg tablet) recommended for *H. pylori* treatment compared to FAM dose (40 mg tablet). First derivative (D_1_/A) could resolve MET and FAM while derivative double divisor ratio spectra (D/DDRS) and applying Fourier Functions (FF/A) to absorbance spectra could resolve the ternary mixture AMX, MET and FAM. As a result, the proposed methods are suggested to be applied in quality control labs for the cited drugs’ analysis either alone or combined. Moreover, it could be applied in further studies investigating the drugs in gastric fluid.

In addition to being straightforward, repeatable, and possessing high sensitivity and selectivity, the outlined spectrophotometric methods also align well with the core principles of green analytical chemistry, which include limited solvent usage, reduced waste production, and quick analysis with low energy usage. This increases the suggested approaches’ level of sustainability. The greenness of the approaches was evaluated using a variety of greenness metrics [34–38]. Besides, the methods were entirely evaluated employing (RGB) method for whiteness assessment [39] and practically appraised employing Blue Applicability Grade Index (BAGI) [40].

## Experimental

### Instrumentation

A PerkinElmer UV/VIS spectrophotometer and a 1-cm quartz cell (Analytik Jena) were used. or weighing, a cal-TEC balance model SPB 31, Germany was used. A Julabo sonicator model USR 3, Julabo Labortechnik, GMBH, Germany was used for sonication. A pH meter was applied to adjust the pH of simulated gastric fluid. All calculations involved in this study were done using Microsoft office Excel 2010 software.

### Materials and reagents

Working standard AMX was supplied from Pharco Pharmaceuticals Company, Alexandria, Egypt. Its purity was 99%. On the other hand, FAM and MET working standard substances were supplied from Medical Union Pharmaceuticals Company, Ismailia, Egypt with purity 99.6% and ≥ 98%, respectively. From the local market, Biomox^R^ tablets (labeled to contain 500 mg AMX per tablet) and Famotak^R^ tablets (labeled to contain 40 mg FAM), both manufactured by SEDICO, Egypt were purchased. Also, Flagyl^R^ tablets (labeled to contain 250 mg MET per tablet), manufactured by SANOFI, Egypt were purchased from the local market. Analytical grade methanol was supplied from Sigma-Aldrich Chemie GmbH, Swizterland Germany.

### General procedure and construction of calibration graphs

#### Preparation of stock and working standard solutions

**Fig. 1:**
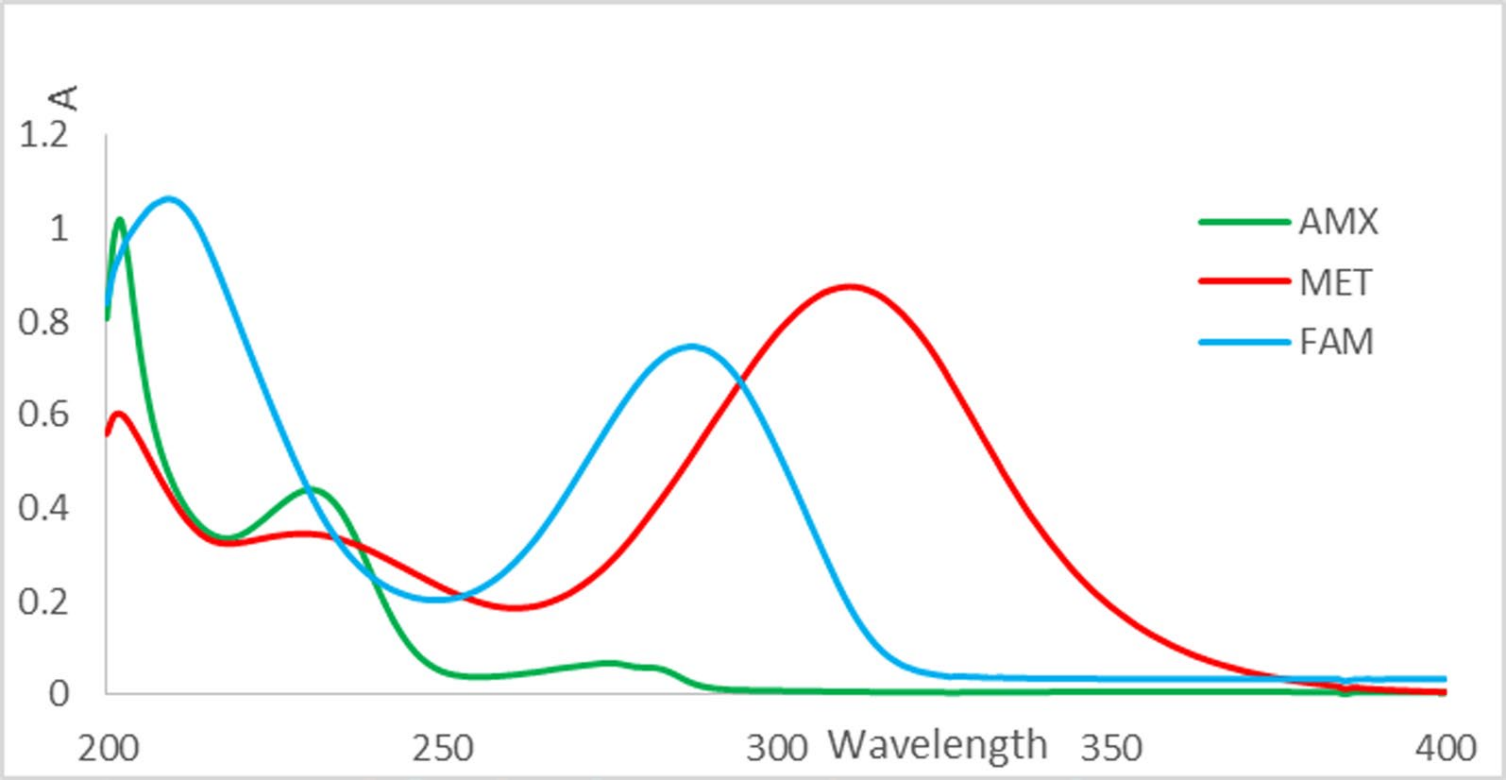
Absorbance spectra of 16 µg mL^−**1**^ AMX ( ), 16 µg mL^−1^ MET ( ) and 15 µg mL^−1^ FAM ( ) in methanol

Analytical grade methanol has been utilized to generate stock solutions of AMX 2000 µg mL^-1^, MET 2000 µg mL^-1^, and FAM 1000 µg mL^-1^, which were then kept out of direct sunlight. Next, suitable aliquots of the stock solutions were diluted with analytical grade methanol into 50 mL volumetric containers to create diluted stock solutions of AMX 80 µg mL^-1^, MET 80 µg mL^-1^, and FAM 60 µg mL^-1^. In order to achieve the target concentration ranges of 12-40, 4-20, and 3-20 µg mL^-1^ for AMX, MET, and FAM, respectively, suitable portions of the diluted stock solutions were diluted with analytical grade methanol and added to a series of 10-mL volumetric flasks to create the final working solutions. The zero order (A) absorption spectra (Fig. 1) of the prepared standard solutions were scanned from 200 to 400 nm at 1 nm interval and stored in the computer. All the stored spectra were manipulated using WinAspect software.

#### Method I: derivative, (D_1_/A) method

**Fig. 2:**
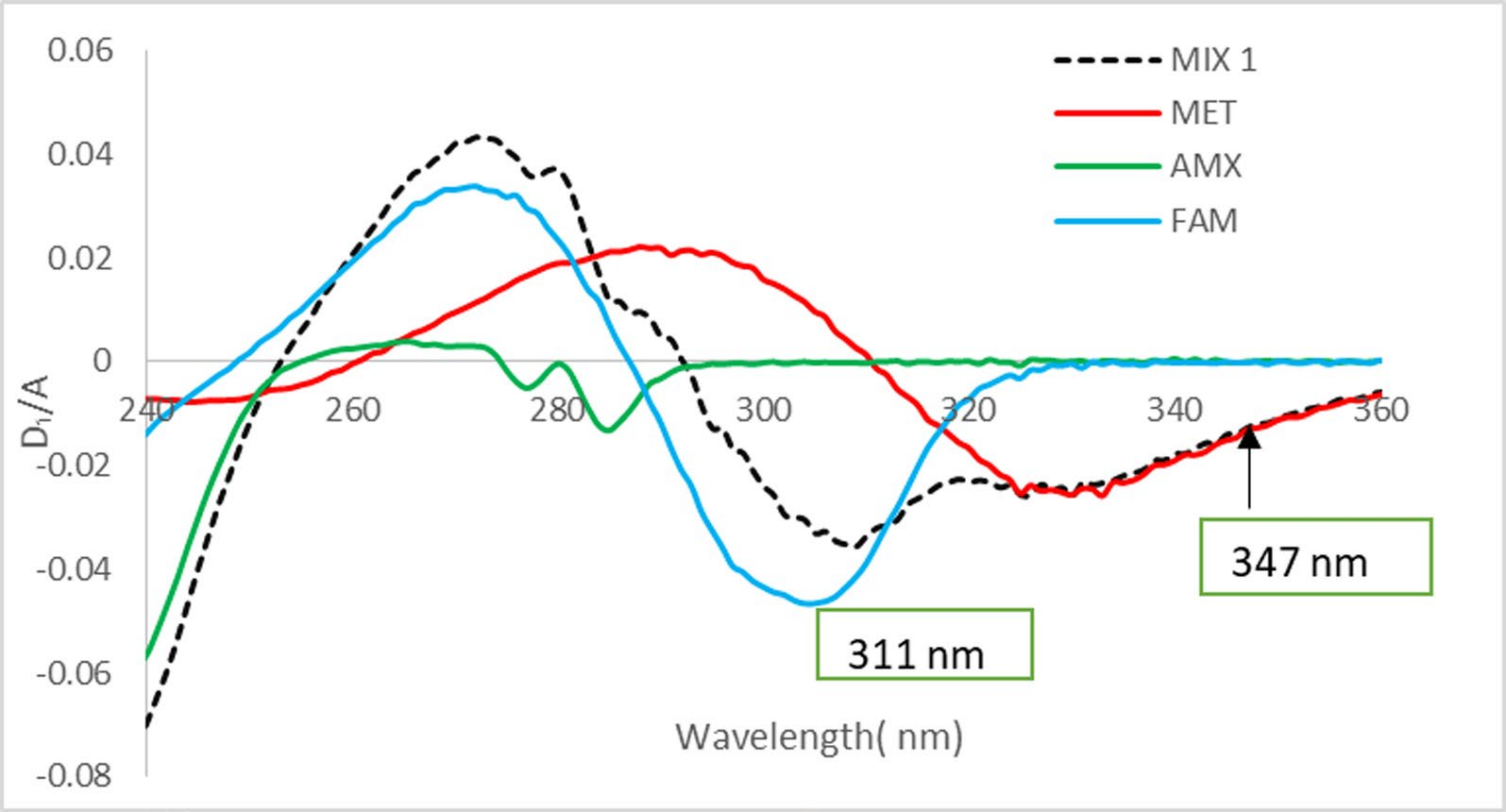
First derivative spectra of a synthetic mixture of the three drugs ( ) & its components 28 µg mL^−1^ AMX ( ), 16 µg mL^−1^ MET ( ) & 20 µg mL^−1^ FAM ( ) in methanol

The D_1_/A spectra were obtained from absorption spectra using 1 nm as a wavelength interval (∆λ=1.) The absolute values of D_1_/A amplitudes at 347 nm (for MET) and 311 nm (for FAM) were plotted against the corresponding concentrations then the regression equation for each of MET &FAM was computed (Fig. 2).

#### Method II: derivative double divisor ratio spectra, (D/DDRS) method

##### For determination of AMX

**Fig. 3:**
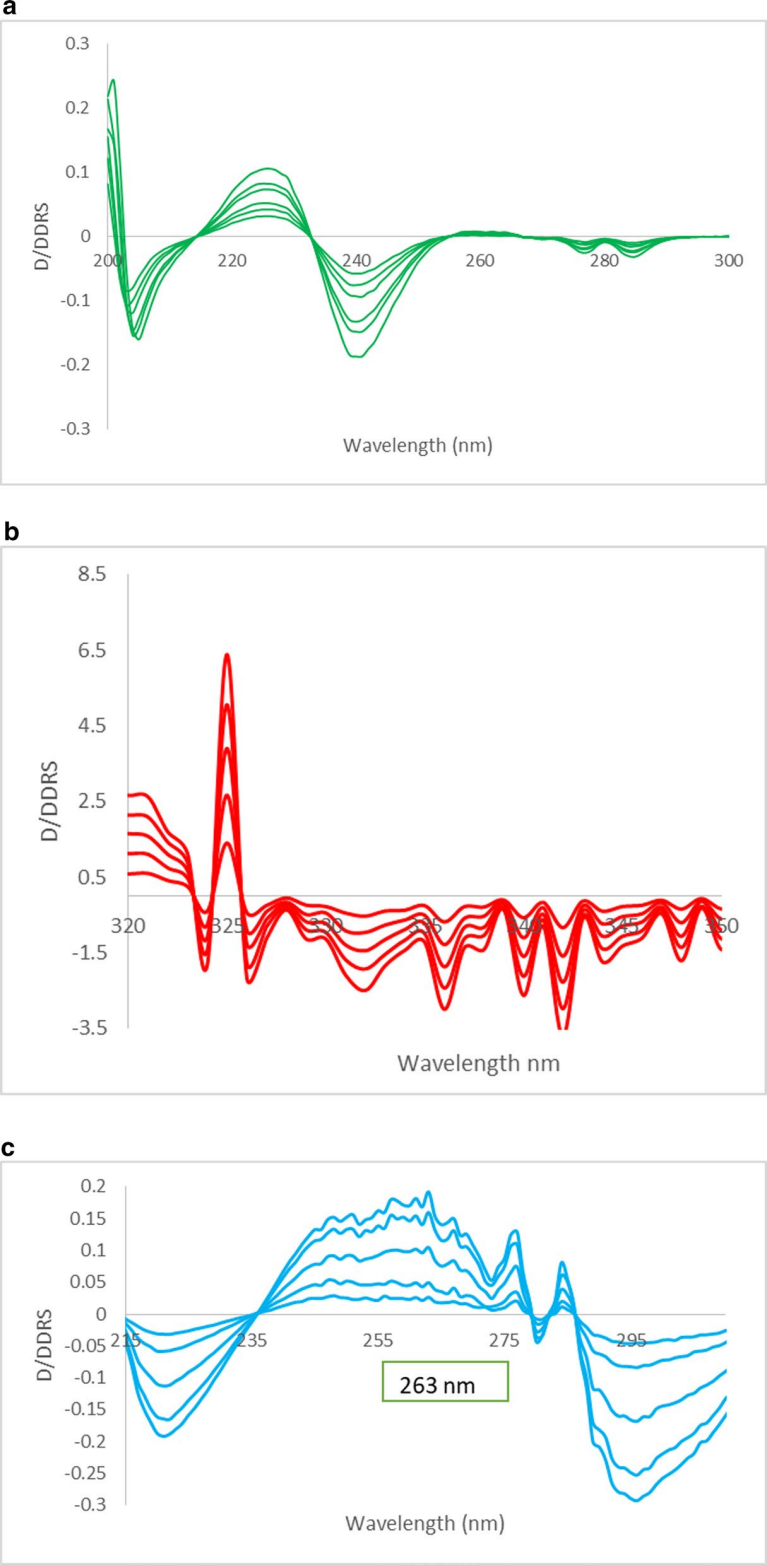
**a** First derivative of the ratio spectra of AMX in methanol at the linearity range (12–40 µg mL^−1^) showing the selected λ to solve AMX (243 nm). **b** First derivative of the ratio spectra of MET in methanol at the linearity range (4–20 µg mL^−**1**^**)** showing the selected λ to solve MET (333 nm). **c** First derivative of the ratio spectra of FAM in methanol at the linearity range (3–20 µg mL^−**1**^) showing the selected λ to solve FAM (263 nm)

The stored absorption spectra of AMX were divided by the sum of the spectra of 12 μg mL^-1^ standard MET solution and 6 μg mL^-1^ standard FAM solution, being taken as the double divisor. Then the created double devisor ratio spectra were saved. The first derivative spectra were obtained. The calibration curve for AMX was set by plotting the absolute D/DDRS values of amplitudes at 243 nm versus the corresponding AMX concentrations then the regression equation was computed (Fig. 3a and Suppl. Fig. S2a).

##### For determination of MET

The same steps of AMX determination were done by dividing the stored absorption spectra of MET by the sum of the spectra of 12 μg mL^-1^ standard AMX solution and 6 μg mL^-1^ standard FAM solution. Then the first derivative spectra were derived. The calibration curve for MET was set by plotting the absolute D/DDRS values of amplitudes at 333 nm versus the corresponding concentrations then the regression equation was derived (Fig. 3b and Suppl. Fig. S2b).

##### For determination of FAM

The same steps of AMX determination were done by dividing the stored spectra of FAM by the sum of the spectra of 12 μg mL^-1^ standard AMX solution and 4 μg mL^-1^ standard MET solution. Then the developed ratio spectra were used to calculate the corresponding first derivative spectra. The calibration curve for FAM was set by plotting the absolute D/DDRS values of amplitudes at 263 nm versus the corresponding concentrations then the regression equation was derived (Fig. 3c and Suppl. Fig. S2c).

#### Method III: fourier functions, (FF/A) method

The Fourier function coefficients (t^´^_j)_ values were calculated for each of AMX, FAM & MET from their stored absorption spectra using eight points combined Fourier function, T´ = [cos x + cos (x + 45º)] at 4 nm interval as follows [23]:

$$t^{\prime}_{j} = \, \left\{ {\left( { + {1}.{7}0{7}} \right){\text{ A}}_{0} + \, \left( { + 0.{7}0{7}} \right){\text{ A}}_{{1}} + \, \left( { - 0.{7}0{7}} \right){\text{ A}}_{{2}} + \, \left( { - {1}.{7}0{7}} \right){\text{ A}}_{{3}} + \, \left( { - {1}.{7}0{7}} \right){\text{ A}}_{{4}} + \, \left( { - 0.{7}0{7}} \right){\text{ A}}_{{5}} + \, \left( { + 0.{7}0{7}} \right){\text{ A}}_{{6}} + \, \left( { + {1}.{7}0{7}} \right){\text{ A}}_{{7}} } \right\} \, /{ 4}$$

where A_0_ to A_7_ stand for eight absorbance values at 4 nm intervals for determination of all of the three drugs. The numbers in brackets are the values of the selected combined Fourier function, T´ = [cos x + cos (x + 45º)].

**Fig. 4:**
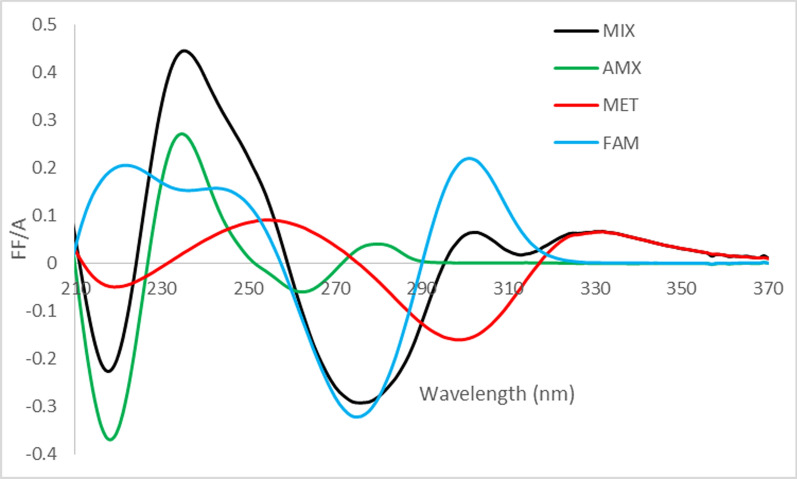
Transformed absorbance spectra of a synthetic mixture of the three drugs () & its components 28 µg mL^−1^ AMX ( ), 16 µg mL^−1^ MET ( ) and 20 µg mL^−1^ FAM ( ) derived using 8- points, [cos x + cos (x + 45º)], combined trigonometric Fourier functions at 4 nm intervals

The resulted convoluted spectra derived for the three cited drugs are shown in Fig. 4. Three wavelengths which are 235 nm, 275 nm & 330 nm have been selected for constructing three simultaneous equations as follows:

1 $${\text{t}}^{\prime}_{{{235}}} =\, \alpha^{\prime}_{{{235}}} {\text{C}}_{{{\text{AMX}}}} + \, {\upbeta}^{\prime}_{{{235}}} {\text{C}}_{{{\text{FAM}}}} + \,\Upsilon^{\prime}_{{{235}}} {\text{C}}_{{{\text{MET}} }}$$

2 $${\text{t}}^{\prime}_{{{275}}} = \, \alpha^{\prime}_{{{275}}} {\text{C}}_{{{\text{AMX}}}} + \, {\upbeta}^{\prime}_{{{275}}} {\text{C}}_{{{\text{FAM}}}} + \, \Upsilon{^{\prime}}_{{{275}}} {\text{C}}_{{{\text{MET}} }}$$

3 $${\text{t}}^{\prime}_{{{33}0}} = \, \alpha^{\prime}_{{{33}0}} {\text{C}}_{{{\text{AMX}}}} + \, {\upbeta}^{\prime}_{{{33}0}} {\text{C}}_{{{\text{FAM}}}} + \, \Upsilon^{\prime}_{{{33}0}} {\text{C}}_{{{\text{MET}} }}$$

where t^´^_235,_ t^´^_275_ & t^´^_330_ are the Fourier functions coefficients of the mixture at λ _m_ = 235 nm, 275 nm & 330 nm, respectively,

α´_235,_ α´_275_ & α´_330_ are constant analogues to absorptivity of AMX at the selected wavelengths.

ß´_235,_ ß´_275_ & ß´_330_ are constant analogues to absorptivity of FAM at the selected wavelengths.

andϒ´_235,_ ϒ´_275_ & ϒ´_330_ are constant analogues to absorptivity of MET at the selected wavelengths.

Since there is no interference from either AMX or FAM at 330 nm, each of α´_330_ & ß´_330_ = 0. Therefore, Eq. (3) can be rewritten to find the concentration of MET nm as follows:

4 $${\text{t}}^{\prime}_{{{33}0 }} = \, \Upsilon^{\prime}_{{{33}0}} {\text{C}}_{{{\text{MET}} }}$$

Then substituting for MET concentration in Eqs. 1&2 and solving two simultaneous equations for determination of AMX and FAM.

### Method applications

#### Application to individual tablets

Biomox^R^ 500 mg, Famotak^R^ 40 mg and Flagyl^R^ 250 mg were used to individually analyze AMX, FAM and MET, respectively. Weighing and powdering 20 tablets of them individually was done followed by taken accurate individual weights of 500 mg of AMX, 250 mg of FAM and 500 mg of MET into 250 -mL volumetric flasks. A thirty-minute sonication followed by volume made up using methanol to obtain 2000, 1000 and 2000 (µg mL ^−1^) stocks of AMX, FAM and MET, respectively.

After filtration, as described in Sect. "Preparation of stock and working standard solutions", eighty, sixty and eighty (µg mL ^−1^) diluted solutions of these stocks were prepared for AMX, FAM and MET, respectively. Further dilution using 10-mL volumetric flasks. Then the procedure, in Sect. "General procedure and construction of calibration graphs", was followed as described.

#### Application to combined tablets

The above mentioned diluted stocks were used to prepare two solutions of AMX, FAM and MET tablet- mixture: the first dilution reached 40 µg mL^−1^ AMX, 3 µg mL^−1^ FAM and 20 µg mL^−1^ MET (representing a *H.pylori* treatment dosage ratio), the second dilution reached 28 µg mL^−1^ AMX, 20 µg mL^−1^ FAM and 16 µg mL^−1^ MET (representing varied drugs ratios for testing the method applicability). Then the procedure, in Sect. "General procedure and construction of calibration graphs", was followed as described.

#### Application to combined tablets in imitated stomach juice

A liquid similar to the gastric fluid was prepared as described in literature [41, 42]. Following the procedure in Sect. "Application to individual tablets"., the same was done using the generated simulated stomach juice to obtain the same stocks. Again dilution of these stocks was done using methanol according to Sect. "Application to individual tablets", yielding, for AMX, FAM and MET, respectively, 80, 60, and 80 (µg mL^−1^). The same two solutions of AMX, FAM and MET, tablet-mixture listed in Sect. "Application to combined tablets", were prepared and the identical process as described in Sect. "General procedure and construction of calibration graphs" was used.

## Results and discussion

### Derivative, (D_1_/A) method

Figure 1 displayed the UV absorption spectra in zero order for FAM, AMX and MET in methanol spanning the 200–400 nm range.

The overt spectral overlapping of the three compounds hindered the utilization of the standard UV spectrophotometric methods for quantifying them in mixtures.

However, many bands of good amplitude were obtained upon recording D_1_/A UV spectra, which facilitated the identification and determination of MET and FAM more selectively. As Fig. 2 displayed, only one point was employed for the determination of each of FAM and MET. The points in question were decided according to the best obtained values for recovery and RSD%.

Upon quantification of MET, optimal recoveries and RSD% values obtained using the D_1_/A value at 347 nm (zero-crossing of AMX and FAM). FAM could also be determined without any interference from AMX or MET by using the D_1_/A value at 311 nm (zero-crossing of AMX and MET). However, neither D_1_ nor D_2_ succeeded to determine AMX as no zero-crossing points were found for its determination in the presence of the other drugs (Figs. 2& Suppl. Fig. S3). As a consequence, D/DDRS technique was examined for the study of each of the above mentioned drugs while the other two were present.

Upon trying ∆λ1, ∆λ2, ∆λ4 and ∆λ6, the D_1_/A curves got smoother but ∆λ1 gave acceptable details with the best accepted recoveries at the selected wavelength for each drug.

### Derivative double divisor ratio spectra, D/DDRS method

D/DDRS was investigated in order to analyze FAM, AMX and MET when present in their ternary mixtures. This method is favored over the zero-crossing derivative method since it does not rely on a single point with possible experimental shift, instead the working wavelengths constitute the range of coincidence between the mixture and the drug of interest. One of the weaknesses of the zero-crossing approach is the possibility of tiny wavelength drifts, which could impair the method's repeatability and cause the working wavelengths to diverge from the peaks. [43].

For D/DDRS method, the optimization of double divisor strength is an important experimental parameter since it affects the values of the ratio derivative.

The double divisor concentration was optimized. The ideal balance of linearity (Suppl. Table S1), sensitivity (Suppl. Table S2) and repeatability (Suppl. Table S3) was obtained upon conducting the investigation at the candidate wavelengths, using 12 μg mL^−1^ MET and 6 μg mL^−1^ FAM (for AMX), 12 μg mL^−1^ AMX and 6 μg mL^−1^ FAM (for MET) and 12 μg mL^−1^ AMX and 4 μg mL^−1^ MET (for FAM) as double divisors.

By dividing each discrete AMX standard's ratio spectrum by the total of 12 μg mL^−1^ MET and 6 μg mL^−1^ FAM, the ratio spectra of the standards at increasing concentrations in methanol were obtained and shown in (Suppl. Fig. S2a). The first ratio derivative spectra were computed and AMX could be ascertained by using the amplitude value of the spectrum at 243 nm in the absence of interference from neither MET nor FAM (Fig. 3a).

Upon the determination of MET, a similar procedure was adopted by dividing each concentration spectrum by the sum of the spectrum of 12 μg mL^−1^ AMX and 6 μg mL^−1^ FAM as shown in Suppl. Fig. S2b. The working wavelength of 333 nm was employed to ascertain MET without interference from neither AMX nor FAM (Fig. 3b) after the computation of the first ratio derivative spectra.

For the determination of FAM, the same technique was followed, by dividing each concentration spectrum by the sum of the spectrum of 12 μg mL^−1^ AMX and 4 μg mL^−1^ MET as shown in Suppl. Fig. S2c. FAM was quantified at the wavelength of 263 nm without interference from neither AMX nor MET (Fig. 3c) following the computation of the first ratio derivative spectra.

As D_1_/A method, different wavelength intervals were tried, ∆λ1, 2, 4 and 6, and ∆λ1 gave the best accepted recoveries at the selected wavelength for each drug. Then it was applied to FAM, AMX and MET mixture as shown in the following representative examples (Suppl. Fig. S4-S6).

### Fourier functions (FF/A) method

In this study, the absorbance spectra of AMX, MET and FAM show significant overlap. Moreover, FAM has a low concentration relative to AMX and MET in the selected dosage regimen for *H. pylori* treatment [3–6]. Since Fourier Functions correct these mentioned problems, the different parameters associated with the calculation of the Fourier coefficients were optimized [23]. These include the selection of T´ = [cos x + cos (x + 45º)] as the combined trigonometric Fourier function, number of points (8 points) and wavelength interval (4 nm).

This method worked out to generate an exceptionally pure analytical spectra at the candidate points which were 235 nm, 330 nm and 275 nm as shown in Fig. 4.

Equations (1, 2 and 4) were rearranged to compute the strengths of the above-mentioned drugs as follows:

#### For determination of MET

It was determined directly using the following equation:

5 $${\text{C}}_{{{\text{MET}}}} = t^{\prime}_{{{33}0}} /_{ } \Upsilon^{\prime}_{{{3} }}$$

#### For determination of AMX

It was determined using the following equation:

6 $$ {\text{C}}_{{{\text{AMX}}}}  = {\text{ t}}_{{235}}^{\prime } \,{{\upbeta}}_{{275}}^{\prime }  - {\text{ t}}_{{275}}^{\prime } {{\upbeta}}_{{235}}^{\prime }  + {\text{ C}}_{{{\text{MET}}}} \Upsilon _{{275}}^{\prime } {{\upbeta}}_{{235}}^{\prime }  - {\text{ C}}_{{{\text{MET}}}} \Upsilon _{{235}}^{\prime } {{\upbeta}}_{{275}}^{\prime } /{{\upbeta}}_{{275}}^{\prime } \alpha _{{235}}  - \alpha _{{275}}^{\prime } {{\upbeta}}_{{235}}^{\prime }  $$

#### For determination of FAM

It was determined using the following equation:

7 $${\text{C}}_{{{\text{FAM}}}} = {\text{ t}}^{\prime}_{{{275}}} \alpha^{\prime}_{{{235}}} {-}{\text{ t}}^{\prime}_{{{235}}} \alpha^{\prime}_{{{275}}} + {\text{ C}}_{{{\text{MET}}}} \Upsilon^{\prime}_{{{235}}} \alpha_{{{275}}} - {\text{ C}}_{{{\text{MET}}}} \Upsilon^{\prime}_{{{275}}} \alpha^{\prime}_{{{235}}} /{{\upbeta}}^{\prime}_{{{275}}} \alpha^{\prime}_{{{235}}} {-} \, \alpha^{\prime}_{{{275}}} {{\upbeta}}^{\prime}_{{{235}}}$$

Then it was applied to FAM, AMX and MET mixture as shown in the following representative examples (Suppl. Fig. S7).

### Validation of the proposed methods

In compliance with ICH guidelines, the established methods' validation was completed [44].

#### Linearity

For each proposed method, the linearity (using five concentrations) of AMX, MET and FAM described in Table 1 displayed strong correlation coefficients (r ≥ 0.9999) and tiny intercepts, revealing that the calibration graphs' linearity was adequate [45].

**Table 1:** Analytical parameters for determination of AMX, MET and FAM ternary mixture using the proposed spectrophotometric methods

Parameters	Method I D_1_/A method		Method II D/DDRS method			Method III FF/A method		
	MET	FAM	AMX	MET	FAM	AMX	MET	FAM
Wavelength (nm)	347	311	243	333	263	235	330	275
Concentration range ( µg mL^−1^)	4–20	3–20	12–40	4–20	3–20	12–40	4–20	3–20
Intercept (a)	7.00 × 10^–4^	7.00 × 10^–4^	7.00 × 10^–4^	3.59 × 10^–2^	5.40 × 10^–3^	4.50 × 10^–3^	2.30 × 10^–3^	3.00 × 10^–4^
Sa^a^	2.59 × 10^–4^	3.50 × 10^–4^	1.04 × 10^–3^	3.52 × 10^–2^	2.26 × 10^–3^	8.92 × 10^–4^	2.14 × 10^–4^	1.27 × 10^–3^
Slope (b)	8.31 × 10^–4^	1.64 × 10^–3^	4.10 × 10^–3^	9.42 × 10^–2^	9.20 × 10^–3^	9.50 × 10^–3^	4.10 × 10^–3^	1.65 × 10^–2^
Sb^b^	1.65 × 10^–5^	1.27 × 10^–5^	4.14 × 10^–5^	1.89 × 10^–3^	1.66 × 10^–4^	3.54 × 10^–5^	1.62 × 10^–5^	9.43 × 10^–5^
RSD% of slope	1.99	0.77	1.01	2.01	1.80	0.37	0.40	0.57
Correlation coefficient (r)	0.9999	0.9999	0.99995	0.9999	0.9999	1	1	0.9999
Sy/x^c^	2.47 × 10^–4^	3.84 × 10^–4^	9.18 × 10^–4^	3.36 × 10^–2^	2.48 × 10^–3^	7.85 × 10^–4^	2.04 × 10^–4^	1.39 × 10^–3^
F^d^	9752.84	16,783.72	60,671.27	6788.87	11,703.53	72,711.89	64,657.29	30,747.43
Significance F	1.03 × 10^–4^	5.96 × 10^–5^	1.65 × 10^–5^	1.47 × 10^–4^	8.54 × 10^–5^	1.12 × 10^–7^	1.34 × 10^–7^	4.09 × 10^–7^
LOD^e^ (μg/mL)	1.02	0.77	0.74	1.18	0.89	0.38	0.72	0.55
LOQ^f^ (μg/mL)	3.09	2.34	2.24	3.57	2.70	1.17	2.17	1.67

^a^Standard deviation of the intercept ^b^Standard deviation of the slope ^c^Standard deviation of residual ^d^ Variance ratio, equals the mean of squares due to regression divided by the mean of squares about regression (due to residuals) ^e^Limit of detection. ^f^Limit of quantification

Linear least squares treatment of AMX, MET and FAM linearity results was adopted. The value of the RSD% of the slope S_b_ did not outstrip 2% suggesting fine linearity. The small values of significance F verified the small degree of scattering AMX, MET and FAM experimental data points around the regression line as well.

#### Detection and quantification limits

The formulae were used to determine both LOD and LOQ in accordance with ICH recommendations. Multiplying the following formulae: σ/b by 3.3 and by 10 for LOD and LOQ computation, respectively, taking into consideration that σ refers to response standard deviation and b is the slope. Low LOD and LOQ were reached for each medication using the outlined methods, and these results were verified through experimentation (Table 1).

#### Accuracy and precision

The investigation of accuracy involved examining the presence of AMX, MET, or FAM in three varied concentration levels synthetic combinations (Tables S4 and S5). Computation of mean recoveries and Er% was done as seen in Table S4 and Table S5).

Then it was calculated from the obtained results as the mean of recoveries of the assayed analyte. The excellent recoveries obtained, and low percentage error indicated the high accuracy of the proposed methods.

To ensure precision, the assessments of three concentration levels of AMX, MET, or FAM were done thrice on the same day (intra-day) and three days apart. Then the relative standard deviations were computed, as shown in table S4 and table S5.

The findings of accuracy with intra and inter-day precision experiments clarified that Er% and RSD% were less than 2%, signaling an excellent level of accuracy and precision for the proposed approaches.

#### Specificity

Method specificity was investigated by the analysis of three synthetic mixtures at various concentration ratios within the working range of either AMX, MET or FAM and two different mixture solutions of their individual commercial tablets. The % recovery, RSD%, and Er% values were sufficient to highlight the great specificity of the techniques suggested for either AMX, MET, or FAM simultaneous analysis in the existence of the others and tablet excipients (Table S4, S5).

#### Stability of solutions

The stock and the diluted stock methanolic solutions of FAM and MET were stable and can be stored in refrigerator for at least a weak yet those of AMX were freshly prepared due to their instability. It was confirmed that sample solutions and standard working solutions were stable during handling; no appreciable spectrophotometric alterations were observed for a minimum of six hours.

### Applications of the proposed methods

#### Assay of FAM, MET and AMX in their marketed tablets

The suggested UV spectrophotometric techniques were employed for analyzing marketed tablet dosage formulations.; each of which for its contained drug; FAM in Famotak® 40 mg, MET in Flagyl® 250 mg and AMX in Biomox® 500 mg. The relevant calibration graph for every drug was utilized to generate recovery data, and the results, which were 99.13 ± 0.62 and 98.90 ± 1.13 for MET and FAM, respectively by D_1_/A method (n = 3), 100.85 ± 1.20, 99.70 ± 0.30 and 99.33 ± 1.55 for AMX, MET and FAM, respectively by D/DDRS method (n = 3) and 98.25 ± 1.07, 99.36 ± 0.58 and 99.40 ± 1.23 for AMX, MET and FAM, respectively by FF/A method were excellent.

#### Assay of FAM, MET and AMX in laboratory-prepared combined tablet mixtures

As part of an *H. pylori* treatment regimen, tablets containing all three medications given together. Analytical grade methanol was used for the preparation of combined- tablet mixture solutions which were quantified utilizing the proposed UV spectrophotometric methods. Excellent outcomes were attained (Suppl. Table S6). The analyses of the combined -tablet mixture solution by D_1_/A method, D/DDRS and FF/A method are illustrated as representative examples in Suppl. Figs. S8 and S9a, S9b and S9c.

#### Assay of FAM, MET and AMX in laboratory-prepared combined tablet mixtures in a gastric fluid simulation

Combined-tablet mixture solutions were prepared in imitated gastric juice for the suitability investigation of the suggested UV spectrophotometric techniques for simultaneous assessment of the three cited medications upon existence of the stomach fluid.

It´s noteworthy to mention that, simulated gastric fluid didn´t interfere with the analysis. The outcomes were displayed in Table 2. Suppl. Figs. S10 & S11 refer to the analysis of the combined- tablet mixture solution in imitated gastric juice by D_1_/A method and FF/A, respectively.

**Table 2:** Assay results for determination of AMX, MET and FAM by the proposed spectrophotometric methods in their laboratory- prepared combined tablet mixtures in simulated gastric fluid (n = 3)

	Nominal value µg mL^−1^		Method I D_1_/A method				Method II D/DDRS method				Method III FF/A method			
	Mix 1	Mix 2	Mean % recovery ± SD^a^		RSD (%)^b^		Mean% recovery ± SD		RSD (%)		Mean% recovery ± SD		RSD (%)	
AMX	40	28					99.24 ± 0.77	100.13 ± 0.50	0.78	0.50	99.26 ± 0.88	100.13 ± 1.04	0.89	1.04
MET	20	16	99.13 ± 0.29	98.70 ± 0.53	0.29	0.54	101.30 ± 1.17	99.70 ± 0.86	1.15	0.86	100.05 ± 1.69	99.27 ± 0.30	1.69	0.30
FAM	3	20	98.93 ± 1.12	100.44 ± 1.20	1.13	1.19	100.20 ± 0.95	101.16 ± 0.34	0.95	0.34	99.87 ± 0.25	99.97 ± 1.18	0.25	1.18

^**a**^ Mean ± SD of three determinations

^**b**^ % Relative standard deviation

It´s well understood that the metabolism of oral medicines in the gastrointestinal system plays a key part in their bioavailability. In vivo elimination of *H.pylori* is problematic because this pathogenic organism is lodged in the stomach epithelium, where it is difficult for several medicines to get inside at therapeutic concentrations [28]. The investigation of AMX's distribution in the stomach juice is crucial due to its extensive usage in first-line eradication therapy [28]. AMX's amphoteric properties, pKa 2.4 and 7.2, point out that at exceptionally low or high pH values, the molecule's un-ionized form predominates, a chemical form essential for the gastric mucosal surface's enhanced lipid penetration. In order to guarantee the effectiveness of antibiotic intervention in eradication procedures, it is crucial to determine the presence of intact AMX in imitated stomach juice [46, 47]. Conversely, as acid secretion rises, so do MET transfer [48]. FAM is said to reduce stomach acid secretion by inhibiting the effect of histamine, which makes it useful in the management of gastritis.

### Statistical comparison

A comparison in statistical terms was established between the outcomes of the assay for FAM, AMX, and MET in the mixed tablet combinations that were made in the lab gathered through the suggested spectrophotometric methods and the authors' HPTLC report [28] using the test of one way of analysis of variance (ANOVA) [44] for FAM, AMX and MET, Suppl. Table S7.

The Comparison of recovery data generated by over two analytical methods is appropriately conducted using the ANOVA test. There were no appreciable variations amongst the suggested approaches, as indicated by the computed F values which were below the critical value.

#### Greenness evaluation of the proposed UV-spectrophotometric methods

Hazard-free environment is the target of Green Analytical Chemistry. Since analytical laboratories have an impact on the environment, various methodologies have been established in order to appraise how environmentally friendly analytical procedures are.

First, the proposed UV spectrophotometric methods' greenness was evaluated using Energy Saving Analyses (ESAs). This was accomplished by figuring out the overall penalty points depending on the kind of reagent and the solvents utilized, as well as the equipment employed in terms of energy consumption, workplace risks, and waste generated throughout the study. The ESA score was 94 (Table 3) which indicates excellent green analysis [34].

**Table 3:** Assessment of the proposed spectrophotometric methods using different greenness metrics

Eco-scale assessment		GAPI	NEMI	AGREE
Reported HPLC method [26]				
Reagents	Penalty points			
Disodium hydrogen phosphate	0			
Acetonitrile	1 × 4 = 4			
Instrument				
Energy	1			
Occupational hazard	0			
Waste	3			
Total penalty points	∑ 5			
Eco- scale assessment score	100–8 = 92			
Proposed spectrophotometric methods				
Reagents	Penalty points			
Methanol	1 × 6 = 6			
Instrument				
Energy	0			
Occupational hazard	0			
Waste	0			
Total penalty points	∑ 6			
Eco- scale assessment score	100–6 = 94			

Then a new tool called GAPI, firstly used in 2018 [35], was adopted assessment of the suggested UV-spectrophotometric techniques' greenness. The five pentagrams that make up the GAPI tool are colored from green through yellow to red, with red denoting the most severe environmental impact. This tool measures how environmentally friendly each stage of a whole analytical process is. Nevertheless, a recent tool and software, called modified GAPI (MoGAPI) were introduced. Alongside supplying the distinctive pictograms of the ordinary GAPI tool, this software calculates a total rank to provide a comprehensive assessment of the method's greenness Thus, it enables sorting through analytical approaches based on their ultimate ranks [36]. With one red region, six yellow and eight green regions, the resulting GAPI pictogram pointed out that the suggested UV-spectrophotometric procedures were very green (Table 3). Region one is red emphasizing the offline sample preparation. The following are denoted by the six yellow regions: regions five and six are the use of straightforward techniques and micro-extraction, respectively; region seven is utilizing environmentally friendly solvents; region nine represents solvent volume from10 till100 ml; zone ten is mildly hazardous solvents utilization; and region eleven is combustibility measure of two or three on its scale. Finally, the remaining eight green regions indicated the absence of the following: preservatives (region 2), transfer (region 3), storing (region 4), sophisticated care such as derivatization (region 8), energy consumption falls below 0.1 kilowatt/ sample (region 12), the use of closed system (region 13), the waste quantity is below 1 mL (region 14) as well as waste treatment (region 15). Besides, the proposed methods are quantitative as reflected by the oval shape at the center of region five. Nonetheless, yellow IVa and IVb subsectors refer to the utilization of methanol which is a moderately toxic reagent with NFPA inflammability score 3. The three green subsectors, Va, Vb and Vc signify common instrumentation setup, energy consumption below 0.1 kilowatt/ sample and the use of closed system. White color was given to the subsectors that indicate inapplicable criteria. Assigning ultimate ranking of 81 to the proposed method reflects the outstanding greenness of the supposed methods.

Additionally, the suggested UV-spectrophotometric approaches' greenness has been measured using the NEMI tool. The four segments that make up the NEMI pictogram denote the subsequent prerequisites: the procedure must not produce a significant quantity of waste (above fifty grams per sample analysis), and the reagents used must not be persistent, bioaccumulative, or toxic (PBT), hazardous or corrosive. When the designated condition for each quadrant is met, it is colored green; otherwise, it is left blank. [37]. However, because methanol was utilized in the described techniques, the region depicting the technique's dangerous possibility remained empty (Table 3).

Finally, the latest AGREE software that was introduced in 2020 provides a more comprehensive presentation of the method greenness. It is a downloadable calculator that evaluates the method greenness based on the 12 GAC principles (SIGNIFICANCE). It is composed of a circular pictogram of twelve portions with an overall score ≥ 0 ≤ 1 appears in the middle. As the score approaches the unity, the method is greener [38]. Regarding the proposed UV-spectrophotometric methods, the two yellow sections in the perimeter referred to the offline analysis (portion 1) and the biological roots of certain chemicals (portion 10). Portion 11 was colored orange as a small volume of toxic reagent was used. Offline device orientation rendered portion 3 to be colored in red. The green color of the remaining eight portions suggested the compliance with the next GAC requirements, the analysis of 2 ml sample (portion 2), a small number of sample preparation processes (portion 4), a semi- automatic method with limited sample preparation (portion 5), absence of derivatization (portion 6), no waste produced/ run (portion 7), 3 compounds and 21 samples were analyzed each run (portion 8), consuming energy as being a UV- spectrophotometry (portion 9) as well as The extent of combustibility and corrosion of the utilized chemicals (portion 12). The suggested method achieved an overall ranking of 0.70 indicating a good ecofriendly method.

However, as the two compared methods had identical NEMI pictograms (Table 3), the NEMI tool was the least prosperous at giving insight into the analytical methods, despite its simplicity. Reliable numerical evaluations were offered by AGREE and ESA, with varying total scores that were, respectively, out of 100 and 1. Regarding automation and identifying the areas where analytical methods are lacking and require additional greenness enhancements, AGREE outperforms ESA. GAPI and AGREE offer highly expressive colored pictograms [49]. GAPI method is regarded as credible as it provides a thorough ecological evaluation of the entire analytical process, from sample preparation to the final analysis. The primary drawback of GAPI is complexity with regard to NEMI and ESA. AGREE is advantageous over GAPI in terms of simplicity and automation [50].

To wrap it up, the proposed spectrophotometric method's greenness was measured using the above mentioned four appraising systems: ESA, NEMI, MoGAPI, and AGREE providing a clear and complementary indication of the method's supremacy over the HPLC technique that was mentioned earlier [26]. (Table 3).

### Assessment of whiteness for the provided spectrophotometric techniques

Recently, the term of White Analytical Chemistry (WAC) has been adopted in the field of analytical methods evaluation. Remarkably, WAC looks at issues of greenness (green) in addition to analytical (red) and practical (blue) variables that affect the method's quality [39].

Accordingly, this Red–Green–Blue (RGB) model uses colors to present the compliance of the investigated methods to the three pillars to be considered during the method’s development.

Lately, this model was simply applied using Excel software which was adopted in this research to appraise the whiteness of the suggested spectrophotometric techniques in comparison with the selected reported techniques [28, 29]

Suppl. Table S8 & Suppl. Fig. S12 showed that the proposed UV- spectrophotometric method outperformed an HPTLC method of the three cited drug and an HPLC method of amoxicillin, metronidazole and vonoprazan. The % whiteness of the three methods were 96.3%, 93.3% and 89.2%, respectively.

To conclude, method validation is important as well as method greenness and applicability/cost- effectiveness which represent an important concept covered by calculating the method’s whiteness.

### Blueness evaluation of the proposed UV- spectrophotometric methods

This section discusses the exploitation of a novel simple index called, Blue Applicability Grade Index (BAGI) appraising the analytical techniques practicality. Ten factors are taken into account by BAGI in order to generate a pictogram and a score illustrating how far the analytical techniques are functional and practical. The final score is displayed using a gradient blue color scale, exploiting separate shades of dark blue, blue, light blue, and white to signify the adherence of the approach with the established criteria, ranging from high to non-adherent, correspondingly [40]. Speaking of the proposed UV- spectrophotometric methods, as shown in figure S13, they were unable to determine more than a sample at a time with volume above 1000 μL (2 white sectors). It is clearly known that, UV-spectrophotometry is a quantitative semi-automatic method depends on simple instrumentation available in most labs, UV spectrophotometer, which was adopted here to quantify three analytes of different chemical classes, resulting in four blue sectors. However, the remaining four dark blue sectors emphasize the method ability to determine beyond 10 samples per hour using methanol as a prevalent commercially- available solvent, with neither extensive sample preparation nor preconcentration. The ultimate assigned BAGI score of 75.0 indicate the success of the supposed methods´ practically. No doubt that, BAGI index is supplementary to green analytical tools.

## Conclusion

The determination of AMX, FAM and MET, using straightforward, accurate, and ecologically safe spectrophotometric techniques was covered in the current study. These are, as far as we are aware, the first spectrophotometric techniques for the simultaneous measurement of the three medications.

In compliance with ICH specifications, the developed techniques were successfully verified and used to analyze AMX, FAM and MET, individually and laboratory-prepared mixed tablet combinations as well as in imitated stomach juice with no interference.

This study could potentially be employed for regular quality control of these three pharmaceuticals when co-formulated in single dosage forms in the coming decades. Moreover, it could be applied in future studies of the three drugs in gastric fluid.

The research future vision is to adjust the method to analyze AMX, FAM and MET in real gastric fluid samples and/or plasma samples for more clinical investigations about this triple therapy action. Also, the proposed chemometric methods which were performed using Excel software could be incorporated in future in an automated computer program that could be incorporated with the software of the spectrophotometric instrument itself for the ease and the automation of the proposed chemometric-UV method to be applied. We are looking forward to fulfilling these aspects in future research.

## Supplementary Information

Supplementary Material 1.

## Data Availability

Data is provided within manuscript and in supplementary material files.

## Abbreviations

AGREE: Analytical greenness metric
AMX: Amoxicillin
BAGI: Blue applicability grade index
D_1_/A: First derivative
D/DDRS: Derivative double divisor ratio spectra
ESA: Analytical eco-scale assessment
FAM: Famotidine
FF/A: Fourier functions to absorbance spectra
GAPI: Green analytical procedure index
GAC: Green analytical chemistry
H. pylori: Helicobacter pylori
ICH: International Council for Harmonization of Technical Requirements for Pharmaceuticals for Human Use
MET: Metronidazole
NEMI: National environmental method index
WAC: White analytical chemistry
