# Supplementary material for: Green Chemometric—assisted UV spectrophotometric techniques for the analysis of Helicobacter pylori triple therapy: amoxicillin, metronidazole and famotidine in bulk, individual and laboratory- prepared combined dosage forms: application to simulated gastric fluid with comprehensive greenness and whiteness appraisals
Source: BMC Chem. 2025 Feb 19;19(1):44. doi: 10.1186/s13065-025-01387-4 (PMC11841278; doi:10.1186/s13065-025-01387-4)

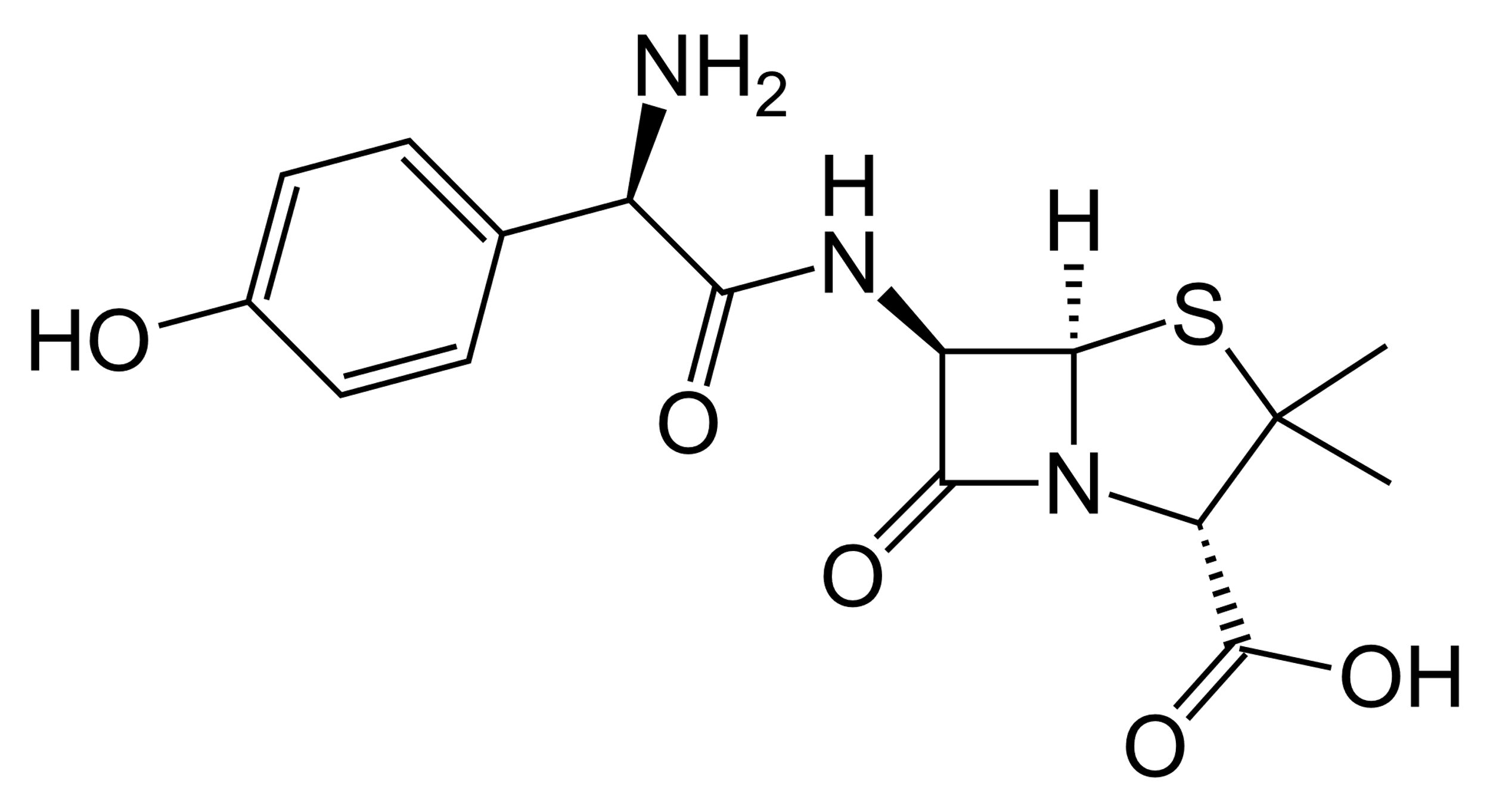

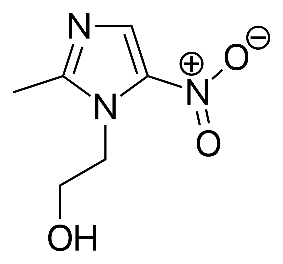

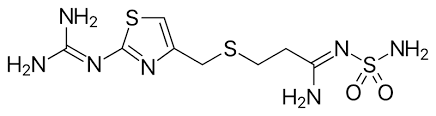


a b c

Figure S1: chemical structures of (a) amoxicillin, (b) metronidazole and (c) famotidine

Figure S2a: Ratio spectra of AMX at different concentrations in methanol at the linearity range (12 – 40 µg mL^-^**^1^ ):** Double divisor (12 µg mL^-^**^1^** MET & 6 µg mL^-^**^1^** FAM)

Figure S2b: Ratio spectra of MET at different concentrations in methanol at the linearity range (4 – 20 µg mL^-^**^1^ ).** Double divisor (12 µg mL^-^**^1^** AMX & 6 µg mL^-^**^1^** FAM )

Figure S2c: Ratio spectra of FAM at different concentrations in methanol at the linearity range (3 – 20 µg mL^-^**^1^ )** Double divisor (12 µg mL^-^**^1^** AMX & 4 µg mL^-^**^1^** MET )

Figure S3: Second derivative spectra of 16 µg mL-1 AMX, 16 µg mL-1 MET and 15 µg mL-1 FAM in methanol. (No zero crossing points where found).

243 nm

Figure S4: Double Divisor ratio spectra of a synthetic mixture of the three drugs and its corresponding concentration of AMX (using 12 µg mL^-1^ MET + 6 µg mL^-1^ FAM as double divisor)

333 nm

Figure S5: Double Divisor ratio spectra of a synthetic mixture of the three drugs and its corresponding concentration of MET (using 12 µg mL-1 AMX + 6 µg mL^-1^ FAM as double divisor)

263 nm

Figure S6: Double Divisor ratio spectra of a synthetic mixture of the three drugs and its corresponding concentration of FAM (using 12 µg mL^-1^ AMX + 4 µg mL^-1^ MET as double divisor)

330 nm

275 nm

235 nm

Figure S7: Transformed absorbance spectra of a synthetic mixture of the three drugs ( ) & its components which are 40 µg mL^-^**^1^** AMX (
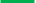
 ), 20 µg mL^-^**^1^** MET ( **
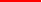
** ) & 3 µg mL^-^**^1^** FAM **(
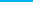
 )** in methanol derived using 8- points, [cos x + cos (x + 45º)], combined trigonometric Fourier functions at 4 nm intervals.

Figure S8: First derivative spectra (D_1_/A) of combined dosage form (…..) & its components which are 40 µg mL^-1^ AMX ( ), 20 µg mL^-1^ MET ( ) & 3 µg mL^-1^ FAM ( ) in methanol

243 nm

Figure S9a: Double Divisor ratio spectra of combined dosage form of the three drugs ( ) and its corresponding concentration of AMX ( ) (using 12 µg mL^-1^ MET + 6 µg mL^-1^ FAM as double divisor)

Figure S9b: Double Divisor ratio spectra of combined dosage form of the three drugs ( ) and its corresponding concentration of MET ( ) (using 12 µg mL-1 AMX + 6 µg mL^-1^ FAM as double divisor)

Figure S9c: Double Divisor ratio spectra of combined dosage form of the three drugs ( ) and its corresponding concentration of FAM ( ) (using 12 µg mL-1 AMX + 4 µg mL-1 MET as double divisor)

Figure S10: First derivative spectra (D_1_/A) of combined dosage form (…..) & its components which are 40 µg mL^-1^ AMX (
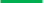
), 20 µg mL MET (
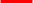
 ) & 3 µg mL^-1^ FAM (
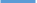
) in simulated gastric fluid

330 nm

275 nm

235 nm

Figure S11: Transformed absorbance spectra of combined dosage form of the three drugs (**___**) & its components which are 40 µg mL^-^**^1^** AMX (
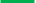
 ), 20 µg mL^-^**^1^** MET ( **
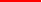
** ) & 3 µg mL^-^**^1^** FAM **(
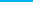
 )** in simulated gastric fluid derived using 8- points, [cos x + cos (x + 45º)], combined trigonometric Fourier functions at 4 nm intervals.


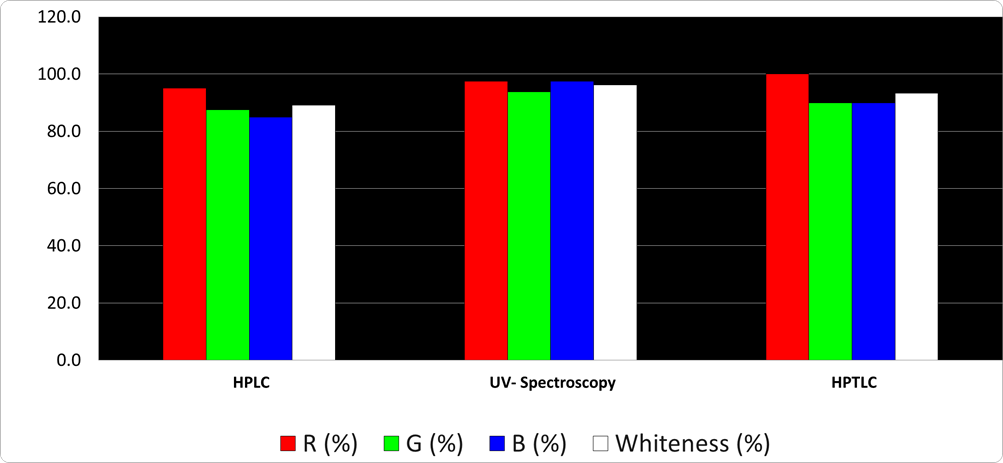


**93.3%**

**89.2%**

**96.3%**

Fig S12: Graphical representation comparing whiteness of the proposed UV- spectrophotometry, HPTLC [28] and HPLC [29] methods

**
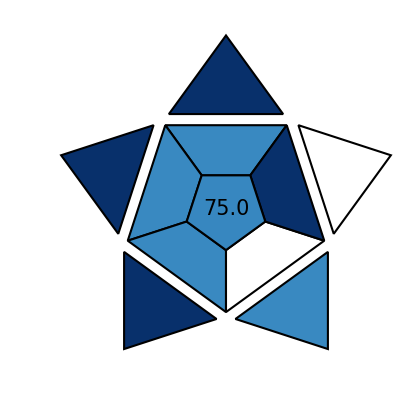
**

Fig S13:  BAGI index pictograms for the proposed UV- spectrophotometric methods.

**Table S1: Influence of divisor concentration on the determination of AMX ***

| **Divisor Conc.**  **(MET+ FAM)**  **(μg/mL)** | **Selected wavelength**  **(nm)** | **a** | **b** | **r** | **Mean % recovery**  **(n=3)** | | |
| --- | --- | --- | --- | --- | --- | --- | --- |
|  |  |  |  |  | **MIX 1** | **MIX 2** | **MIX 3** |
| **4 + 3** | **244** | **0.008** | **-0.0025** | **0.9996** | **86.6** | **84.5** | **95.7** |
| **12 + 6** | \|  \| \| --- \| \| **243** \| | **0.0041** | **0.0007** | **0.99995** | **99.6** | **99** | **98.6** |
| **20 + 6** | **243** | **0.0025** | **-0.0007** | **0.9995** | **91.1** | **91.4** | **98.0** |
| **16 + 18** | **243** | **0.0022** | **-0.0006** | **0.9995** | **91.7** | **92.2** | **98.3** |
| **20 + 18** | **243** | **0.002** | **-0.0006** | **0.9996** | **91.8** | **92.4** | **98.4** |
| **20 + 20** | **243** | **0.0018** | **-0.0005** | **0.9995** | **91.2** | **91.4** | **102** |

*****

**The study was done on three mixtures of AMX: MET: FAM of concentration mix1 (28: 16: 20), mix 2 (16: 16: 18) and mix 3 (40: 20: 3) µg mL^-1^, respectively.**

**Table S2: Influence of divisor concentration on the determination of MET ***

| **Divisor Conc.**  **(AMX + FAM)**  **(μg/mL)** | **Selected wavelength**  **(nm)** | **a** | **b** | **r** | **Mean% recovery**  **(n=3)** | | |
| --- | --- | --- | --- | --- | --- | --- | --- |
|  |  |  |  |  | **MIX 1** | **MIX 2** | **MIX 3** |
| **12 + 3** | **335**  **354** | \| **0.1374** \|  \| \| --- \| --- \|   **0.0627** | **0.0408**  **0.0463** | **0.9995**  **0.9978** | **94.5**  **99.6** | **98.4**  **107.2** | **98.4**  **98.3** |
| **12 + 6** | \| **331**  **333**  **342** \| \| --- \| \|  \| | **0.1012**  **0.0942**  **0.1789** | **0.071**  **0.0359**  **0.1314** | **0.9987**  **0.9999**  **0.9998** | **95.5**  **99.6**  **96.1** | **98.8**  **101.5**  **105.8** | **95.0**  **99.6**  **93.2** |
| **12 + 12** | **298**  **336** | **0.0291**  **0.0124** | **0.0204**  **0.0124** | **0.9999**  **0.9897** | **99.9**  **94.2** | **97.2**  **86.4** | **94.8**  **80.1** |

*** The study was done on three mixtures of AMX: MET: FAM of concentration mix 1 (28: 16: 20), mix 2 (16: 16: 18) and mix 3 (40: 20: 3) µg mL^-1^, respectively.**

**Table S3: Influence of divisor concentration on the determination of FAM ^*^**

| **Divisor Conc.**  **(AMX + MET)**  **(μg/mL)** | **Selected wavelength**  **(nm)** | **a** | **b** | **r** | **Mean % recovery**  **(n=3)** | | |
| --- | --- | --- | --- | --- | --- | --- | --- |
|  |  |  |  |  | **MIX 1** | **MIX 2** | **MIX 3** |
| **12 + 4** | **220**  **263** | **0.0088**  **0.0092** | **0.0043**  **0.0054** | **0.9993**  **0.9999** | **112.3**  **102** | **90.3**  **101.6** | **95.4**  **101.7** |
| **12 + 8** | **257**  **261** | **0.0058**  **0.0062** | **-0.0013**  **-0.0014** | **0.9988**  **0.9985** | **101.1**  **99.2** | **97**  **100.6** | **96.7**  **139.3** |
| **16 + 12** | **259** | **0.0044** | **-0.0013** | **0.9993** | **100.2** | **96.2** | **89.8** |
| **28 + 20** | **259** | **0.0028** | **-0.0008** | **0.9993** | **99.5** | **95.4** | **83.6** |

*** The study was done on three mixtures of AMX: MET: FAM of concentration mix 1 (28: 16: 20), mix 2 (16: 16: 18) and mix 3 (40: 20: 3) µg mL^-1^, respectively.**

**Table S4: Intra-day precision and accuracy for determination of AMX, MET and FAM using the proposed spectrophotometric methods.**

^a^ Mean ± standard deviation for three determinations.

^b^ % Relative standard deviation.

^c^ % Relative error.

| **Method** | **Nominal value**  **(μg mL^-1^)** | | | **Mean %recovery ± SD^a^ (μg mL^-1^)** | | | **RSD (%)^b^** | | | **E_r_(%)^c^** | | |
| --- | --- | --- | --- | --- | --- | --- | --- | --- | --- | --- | --- | --- |
| **Method I**  **Derivative** | **AMX** | **MET** | **FAM** | **AMX** | **MET** | **FAM** | **AMX** | **MET** | **FAM** | **AMX** | **MET** | **FAM** |
|  | **28** | **16** | **20** |  | **98.80 ± 1.12** | **100.30 ±1.27** |  | **1.13** | **1.27** |  | **-1.20** | **0.30** |
|  | **16** | **16** | **18** |  | **100.03 ±1.36** | **99.50 ± 0.74** |  | **1.36** | **0.74** |  | **0.03** | **-0.50** |
|  | **40** | **20** | **3** |  | **99.30 ±0.76** | **100.40 ±0.76** |  | **0.77** | **0.76** |  | **-0.70** | **0.40** |
| **Method II**  **Derivative**  **Ratio** | **28** | **16** | **20** | **99.40 ±0.95** | **99.40 ± 0.97** | **101.70 ±0.35** | **0.96** | **0.98** | **0.35** | **-0.60** | **-0.60** | **1.70** |
|  | **16** | **16** | **18** | **100.20 ±0.98** | **100.30 ±1.12** | **101.50 ± 0.60** | **0.98** | **1.12** | **0.59** | **0.2** | **0.30** | **1.50** |
|  | **40** | **20** | **3** | **99.70 ±1.21** | **98.55 ±0.80** | **101.60 ±0.51** | **1.21** | **0.81** | **0.51** | **-0.30** | **-1.45** | **1.60** |
| **Method III**  **Direct absorbance (MET), Fourier transform convolution(AMX, FAM)** | **28** | **16** | **20** | **100.30 ±0.99** | **99.22 ± 1.96** | **98.38 ±1.50** | **0.99** | **1.98** | **1.52** | **0.30** | **-0.78** | **-1.62** |
|  | **16** | **16** | **18** | **101.26 ±0.15** | **100.98 ±0.69** | **99.75 ± 0.22** | **0.15** | **0.68** | **0.22** | **1.26** | **0.98** | **-0.25** |
|  | **40** | **20** | **3** | **100.25±0.36** | **99.59 ± 1.17** | **100.07± 1.88** | **0.36** | **1.17** | **1.88** | **0.25** | **-0.41** | **0.07** |

**Table S5: Inter-day precision and accuracy for determination of AMX, MET and FAM using the propose spectrophotometric methods**

| **Method** | **Nominal value**  **(μg mL^-1^)** | | | **Mean %recovery ± SD^a^ (μg mL^-1^)** | | | **RSD (%)^b^** | | | **E_r_(%)^c^** | | |
| --- | --- | --- | --- | --- | --- | --- | --- | --- | --- | --- | --- | --- |
| **Method I**  **Derivative** | **AMX** | **MET** | **FAM** | **AMX** | **MET** | **FAM** | **AMX** | **MET** | **FAM** | **AMX** | **MET** | **FAM** |
|  | **28** | **16** | **20** |  | **99.20 ± 0.98** | **101.50± 0.56** |  | **0.99** | **0.55** |  | **-0.80** | **1.50** |
|  | **16** | **16** | **18** |  | **98.95 ±1.13** | **98.90 ± 1.22** |  | **1.14** | **1.23** |  | **-1.05** | **-1.10** |
|  | **40** | **20** | **3** |  | **99.30 ± 0.85** | **100.50 ± 1.11** |  | **0.86** | **1.11** |  | **-0.70** | **0.50** |
| **Method II**  **Derivative**  **Ratio** | **28** | **16** | **20** | **98.80 ± 0.66** | **100.22 ± 1.01** | **101.50 ± 0.55** | **0.66** | **1.01** | **0.54** | **-1.20** | **0.22** | **1.50** |
|  | **16** | **16** | **18** | **100.10 ± 1.37** | **99.20 ± 0.82** | **99.20 ± 0.70** | **1.36** | **0.82** | **0.71** | **0.10** | **-0.80** | **-0.80** |
|  | **40** | **20** | **3** | **100.30 ± 0.85** | **100.92 ± 1.36** | **101.50 ± 0.56** | **0.84** | **1.34** | **0.55** | **0.30** | **0.92** | **1.50** |
| **Method III Direct absorbance (MET), Fourier transform convolution(AMX, FAM)** | **28** | **16** | **20** | **101.30 ± 0.44** | **99.24 ± 1.56** | **100.24 ± 0.97** | **0.43** | **1.57** | **0.97** | **1.30** | **-0.76** | **0.24** |
|  | **16** | **16** | **18** | **100.26 ± 0.18** | **98.22 ± 0.12** | **100.25 ± 0.33** | **0.18** | **0.12** | **0.32** | **0.26** | **-1.78** | **0.25** |
|  | **40** | **20** | **3** | **99.87 ± 0.36** | **99.94 ± 0.83** | **99.14 ± 0.50** | **0.36** | **0.83** | **0.50** | **-0.13** | **-0.06** | **-0.86** |

^a^ Mean % recovery ± standard deviation for nine determinations.

^b^ % Relative standard deviation.

^c^ % Relative error

**Table S6: Assay results for determination of AMX, MET and FAM by the proposed spectrophotometric methods in their laboratory- prepared combined tablet mixtures in methanol (n=3).**

|  | **Nominal value µg mL^-1^** | | **Method I D_1_/A method** | | | | **Method II D/DDRS method** | | | | **Method III FF/A method** | | | |
| --- | --- | --- | --- | --- | --- | --- | --- | --- | --- | --- | --- | --- | --- | --- |
|  |  | |  |  |  |  |  |  |  |  |  |  |  |  |
|  | Mix 1 | Mix 2 | **Mean % recovery ± SD^a^** | | **RSD (%)^b^** | | **% recovery ± SD** | | **RSD (%)** | | **% recovery ± SD** | | **RSD (%)** | |
|  |  | |  | |  |  |  |  |  |  |  |  |  |  |
| **AMX** | **40** | **28** |  | |  | | 101.13 ± 0.81 | 100.17 ± 1.16 | 0.80 | 1.16 | 100.13 ±0.60 | 99.38 ± 1.85 | 0.60 | 1.86 |
| **MET** | **20** | **16** | 99.50 ±0.49 | 100.20 ±0.38 | 0.49 | 0.38 | 100.20 ± 0.70 | 99.40 ± 1.36 | 0.70 | 1.37 | 100.96 ± 0.55 | 100.33 ± 0.72 | 0.54 | 0.72 |
| **FAM** | **3** | **20** | 98.70 ± 0.96 | 100.90 ±1.18 | 0.98 | 1.17 | 98.70 ± 0.96 | 99.20 ± 1.46 | 0.98 | 1.47 | 99.78 ± 1.25 | 98.12 ± 1.80 | 1.25 | 1.83 |

^a^ Mean ± SD of three determinations

**^b^** % Relative standard deviation.

**Table S7: Summary of assay results and one–way analysis of variance (ANOVA) for the determination of AMX, MET and FAM in their laboratory- prepared combined tablet mixtures by the proposed spectrophotometric methods and a reported HPTLC method [28].**

| **% Recovery** | | | | | | | | | | |  |
| --- | --- | --- | --- | --- | --- | --- | --- | --- | --- | --- | --- |
| **Spectrophotometric methods** | | | | | | | | **HPTLC method** | | |  |
| **Method I D_1_/A method** | | **Method II D/DDRS method** | | | **Method III FF/A method** | | |  |  |  |  |
| **MET**  **^1^D at 347 nm** | **FAM**  **^1^D at 311nm** | **AMX ^1^DR at 243 nm** | **MET ^1^DR at 334 nm** | **FAM ^1^DR at 262 nm** | **AMX at 237 nm** | **MET at 341 nm** | **FAM at 309 nm** | **AMX** | **MET** | **FAM** |  |
| 99.50  100.20 | 98.70  100.90 | 101.13  100.17 | 100.20  99.40 | 98.70  99.20 | 100.13  99.38 | 100.96  100.33 | 99.78  98.12 | 100.80  99.20 | 99.00  99.50 | 99.60  98.20 |  |
| **Mean ± SD** | 99.85 ± 0.49 | 99.80 ± 1.56 | 100.65 ± 0.68 | 99.80 ± 0.57 | 98.95 ± 0.35 | 99.76 ± 0.53 | 100.65 ± 0.45 | 98.95± 1.17 | 100 ± 1.13 | 99.25 ± 0.35 | 98.90 ± 0.99 |

|  | **Source of Variation** | **SS** | **df** | **MS** | **F** | **P-value** | **F crit** |
| --- | --- | --- | --- | --- | --- | --- | --- |
| **AMX** | Between Groups | 0.8557 | 2 | 0.42785 | 0.634777 | 0.104131 | 9.552094 |
|  | Within Groups | 2.02205 | 3 | 0.674017 |  |  |  |
|  | Total | 2.87775 | 5 |  |  |  |  |
| **MET** | Between Groups | 1.978537 | 3 | 0.6595512 | 2.969272 | 0.160218 | 6.591382 |
|  | Within Groups | 0.88845 | 4 | 0.222112 |  |  |  |
|  | Total | 2.866987 | 7 |  |  |  |  |
| **FAM** | Between Groups | 1.13 | 3 | 0.376667 | 0.307307 | 0.820111 | 6.591382 |
|  | Within Groups | 4.9028 | 4 | 1.2257 |  |  |  |
|  | Total | 6.0328 | 7 |  |  |  |  |

**Continue table (S7):**

**Table S8: Whiteness evaluation of the proposed spectrophotometric method**


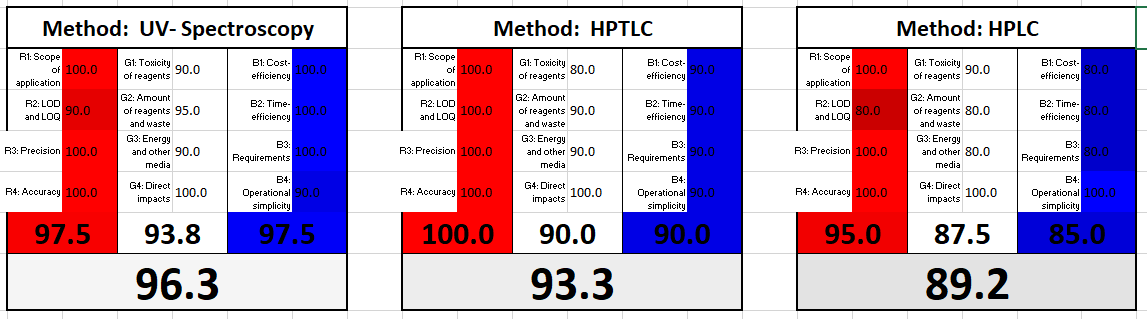

Supplement: Supplementary file 1 — Supplementary Material 1. [file 13065_2025_1387_MOESM1_ESM.docx]
